# Monocyte-to-lymphocyte ratio is significantly associated with positive QuantiFERON-TB Gold-In-Tube and adult survival: an observational study

**DOI:** 10.1038/s41598-022-24376-2

**Published:** 2022-11-27

**Authors:** Hai-bo Hua, Hui-jie Wang

**Affiliations:** Tuberculosis Department, Affiliated Hangzhou Chest Hospital, Zhejiang University School of Medicine, No. 208 East Huancheng Road, Hangzhou, 310003 Zhejiang China

**Keywords:** Infectious diseases, Tuberculosis

## Abstract

This study aimed to find significant factors associated with tuberculosis (TB) infection and disease development. The participants were from National Health and Nutrition Examination Survey (NHANES) and National Death Index (NDI). The tuberculosis infection was defined as a positive QuantiFERON-TB Gold-In-Tube (QFT-GIT). The Least Absolute Shrinkage and Selection Operator (LASSO) model was used to screen variables associated with QFT-GIT among 23 laboratory measures. Then the logistic regression analyses were performed to assess the independent factors, followed by a comprehensive nomogram model construction. Receiver operating characteristic (ROC) and Decision Curve (DCA) analyses were used to assess the performance of comprehensive model on QFT-GIT result and death risk. Of 5256 individuals included, 521 individuals had positive QFT-GIT. LASSO analysis indicated that 11 variables were associated with QFT-GIT result, and logistic regression analyses further found sodium and monocyte-to-lymphocyte ratio (MLR) were independent factors. After adjusting for potential confounders, the correlation of sodium and MLR with QFT-GIT result was still observed. The comprehensive model based on sodium, MLR, and important clinical characteristics can predict 0.8 probability of positive QFT-GIT and achieve more clinical net benefit. ROC analysis by training and validation sets showed the favorable prediction performance. Comprehensive model also presented favorable performance in evaluating the death risk of individuals with positive QFT-GIT. We also found MLR rather than sodium was independently related to the death risk. Both MLR itself and comprehensive model were all significantly related to the positive QFT-GIT and death risk, which might participate in the initiation and progression of tuberculosis infection.

## Introduction

Tuberculosis (TB), caused by mycobacterium tuberculosis (Mtb) infection, is one of the leading causes of death from infectious diseases. World Health Organization (WHO) in 2021 has proposed the “End TB Strategy” to reduce the incidence of TB by 90% between 2015 and 2035^1^. Hence, it needs more effort to realize this target. The related mechanisms of Mtb infection included subversion of expression of key microRNAs (miRNAs) involved in the regulation of host innate and adaptive immune response against Mtb^2^. In addition, the pathological effects also included induction of necrosis, NOD2 signaling, type I interferon production, and autophagy^3^. Latent TB infection and active TB are the two main TB-related statuses. The evolution between TB infection and active TB is multifactorial and the synthesis of ESAT-6 or the induction of alveolar macrophage necrosis are key^4^. About 5–10% of latent TB infection patients will progress to active disease in the first years after primary infection and, despite using the recommended treatment, 20% can still reactivate the infection^5^. Hence, early diagnosis and prevention of latent TB is an important healthcare management strategy to control TB progression.

The strategy for controlling TB has been focused on the development of laboratory tests that can be used to identify those with latent TB who are at risk for developing active TB. The tuberculin skin test (TST) and interferon-gamma release assays (IGRAs) are widely used to evaluate the host immune response and diagnose TB^6^. But their applications are subjected to limitations due to the high cost, insufficient convenience, and technological complexity^7^. Presently, some studies have proposed the use of more basic laboratory measures. Naranbhai et al. found that elevated monocytes to lymphocytes (ML) ratio at 3–4-months old was associated with increased hazards of TB disease before two years among children^8^. They also indicated the significant correlation between ML ratio and TB risk among HIV-infected adults^9,10^. Chedid et al. study indicated that high white blood cell counts and low lymphocyte proportions at baseline were significantly associated with the risk of TB treatment failure^11^. Zhang et al. study suggested that patients with obvious weight loss and relatively lower white blood cell count have a larger TB infection risk^12^. Rees et al. study showed that red blood cell decrease was associated with the development of TB infection^13^. The important role of basic laboratory measures in TB has been paid more and more attention. Although the importance of several laboratory indicators on TB risk has been assessed, it is necessary to find more valuable markers for controlling TB progression. In addition, few studies focused on the prognostic value of laboratory results in TB.

In this study, we used the machine learning LASSO model and logistic regression analyses to detect the influencing factors associated with TB risk among 23 laboratory test results using data from National Health and Nutrition Examination Surveys (NHANES 2011–2012). By matching the corresponding mortality data of samples in the National Death Index (NDI), we further evaluated the prognostic value of important laboratory test results. This study aimed to find significant factors associated with both TB risk and mortality of individuals with TB.

## Methods

### Data source and study population

This study is a cross-sectional analysis to evaluate the important factors associated with TB infection and the survival of individuals. Related information about TB infection and variables were obtained from the National Health and Nutrition Examination Survey (NHANES) (https://www.cdc.gov/nchs/nhanes/). The NHANES interview includes demographic, socioeconomic, dietary, and health-related questions. The examination component consists of medical, dental, and physiological measurements, as well as laboratory tests administered by highly trained medical personnel. The 2011–2012 NHANES cycle included the recent TB testing for all participants ≥ 6 years old. In this study, we excluded the participants under the age of 18 and samples without QuantiFERON-TB Gold-In-Tube (QFT) and tuberculin skin testing (TST) information.

The corresponding survival data of individuals with TB were obtained from National Death Index (NDI) (https://www.cdc.gov/nchs/ndi/) which is linked to the NHANES surveys and contains over 100 million death records. The survey protocol for NHANES data collection is approved by the NCHS institutional review board. The Ethics Committee has confirmed that the data can be analyzed and published with waver of consent from the individuals included. The data used in this study were anonymised before its use.

### Definitions

To determine the TB infection, NHANES participants 6 years of age and older were skin tested with a tuberculin-purified protein derivative (PPD) product, tubersol, a commercially available antigen. Additionally, these NHANES participants were secondarily screened with an FDA-approved IGRA blood test, QuantiFERON-TB Gold In Tube test (QFT-GIT), for TB infection. But the TST has been found to have lower specificity and lower positive predictive value than QFT-GIT. Therefore, in this study we used only the results from the QFT-GIT for the TB infection.

Detailed description of laboratory methodology for QFT-GIT was described elsewhere^14^. The QFT-GIT system uses specialized blood collection tubes, which are used to collect whole blood via venipuncture including a Nil control tube, TB Antigen tube, and a Mitogen tube (positive control)^15^ . The tubes are shaken to mix antigen with the whole blood and incubated at 37 °C + 1 °C for 16 to 24 h^16^. Following the incubation period, plasma is harvested and the amount of IFN-γ produced in response to the peptide antigens is measured by ELISA^17^. Results for the test samples are reported in International Units (IU) relative to a standard curve prepared by testing dilutions of a recombinant human IFN-γ standard^18^.

In this study, individuals were divided into QFT-GIT negative and positive groups. The following criteria were used to interpret the positive QFT-GIT according to the NHANES guidelines, 1) Nil value must be ≤ 8.0 IU gamma interferon (IF)/ml, and 2) TB antigen value minus Nil value must be ≥ 0.35 IU gamma interferon (IF)/ml, and 3) TB antigen value minus Nil value must be ≥ 25% of the Nil value. Individuals with missing or indeterminate QFT-GIT results were excluded. A low response to mitogen (< 0.5 IU/mL) indicates an indeterminate result when a blood sample also has a negative response to the TB antigens.

### Variables

The QFT-GIT result and survival status were chosen as dependent variables. In addition, 36 variables were included in the study. The qualitative variables included gender, age, race, education level, marital status, whether lived in household TB sick person, BMI (kg/m^2^), tuberculin skin test (TST) result, history of TB exposure, application of TB medicine, arthritis classification, smoking status, alcohol use, whether diabetes. TST was performed with 0.1 mL of tuberculin antigen (Tubersol), read by NHANES staff 46–74 h after placement. The positive TST in this study was defined as induration size ≥ 10 mm (commonly used for adults in the US, except for individuals with special risks). Study protocol dictated that at least two separate readers, blinded to each other’s measurements, would measure TST reactions of > 25% of participants. Readers worked in separate rooms and recorded measurements in a computer database; measurements recorded on the first screen were not accessible to subsequent readers.

We also enrolled 23 laboratory test results, including white blood cell count, albumin, sodium, lymphocyte percent, monocyte percent, neutrophils percent, eosinophils percent, basophils percent, lymphocyte number, monocyte number, neutrophils num, eosinophils number, basophils number, red blood cell count, hemoglobin, hematocrit, platelet count, monocyte-to-lymphocyte ratio (MLR), platelets-to-monocyte ratio (PMR), platelets-to-lymphocyte ratio (PLR), neutrophil-to-lymphocyte ratio (NLR), platelets-to-neutrophil ratio (PNR), and prognostic nutritional index (PNI).

### Statistical analysis

We merged the candidate records by corresponding sequence number. Qualitative variables were presented as frequency and the differences between groups were compared by χ^2^ test. Quantitative variables were firstly assessed the normal distribution and presented as means ± standard error (SE). The differences on quantitative variables were compared by Bonferroni test. For all analyses, *P* < 0.05 was considered statistically significant.

We first used the “glmnet” R package to fit the Least Absolute Shrinkage and Selection Operator (LASSO) model to screen latent variables. The QFT-GIT result (positive vs negative) was set as the dependent variable, and all the laboratory test results as independent variables were included in the LASSO model. The ten-fold cross-validation was used to select the penalty term lambda (λ). LASSO analysis minimized the insignificant coefficients to 0 and the nonzero variables were selected for further analysis. We next constructed machine learning model to predict the weight importance of nonzero variables. The model parameters included 1) fold number for cross-validation was 5; 2) maximum iterations was 1000; 3) convergence metric was 0.0001; and 4) alpha was 1.86e-04.

Further, we performed a univariable logistic regression analysis to evaluate the correlation between nonzero variables and QFT-GIT result. Then the variables with *P* < 0.05 in univariable logistic regression analysis were enrolled into the multivariable logistic regression to select the independent factors. Regarding the independent factors, we performed the logistic regression analyses to explore the correlation of these variables with QFT-GIT result after adjusting potential confounder. Further, we constructed a comprehensive nomogram model incorporating above independent factors and significant clinical characteristics to evaluate the possibility for predicting positive QFT-GIT. Decision Curve (DCA) analysis was used to assess the clinical net benefit, and receiver operating characteristic (ROC) analysis in training and validation sets was used to evaluate the prediction performance of comprehensive model. We also performed the ROC and DCA analyses to evaluate the performance of comprehensive model about death risk in individuals with positive QFT-GIT. Finally, the correlation between independent factors and death risk among individuals with positive QFT-GIT was assessed by logistic regression analyses with adjustment of potential confounders.

## Results

### Baseline characteristics of individuals

The NHANES 2011–2012 cycle contained 9756 samples. A total of 5256 participants aged 18–85 years who had QFT-GIT results were included in this study. Among them, 521 (9.9%) individuals had positive QFT-GIT results. The baseline sociodemographic characteristics stratified by QFT-GIT status were shown in Table 1.

**Table 1:** The baseline characteristics of qualitative variables stratified by age and QFT-GIT results.

Covariates	18–34 year			35–64 year			65–85 year		
	Negative (*n* = 1547)	Positive (*n* = 62)	P	Negative (*n* = 2272)	Positive (*n* = 298)	P	Negative (*n* = 915)	Positive (*n* = 162)	P
**Gender**			0.091			0.013			0.005
Male	779	38		1077	164		443	98	
Female	768	24		1195	134		472	64	
**Race**			0.085			< 0.001			< 0.001
Mexican American	179	13		244	50		36	15	
Other Hispanic	157	9		219	49		79	33	
Non-Hispanic White	499	14		843	27		510	34	
Non-Hispanic Black	418	13		622	68		203	44	
Other Race	294	13		344	104		87	36	
**Education level**			0.018			< 0.001			< 0.001
Less than high school	167	14		506	95		282	82	
High school	249	13		475	64		213	29	
More than high school	863	29		1291	139		418	51	
**Marital status**			0.138			0.055			0.828
Married	382	24		1284	182		473	82	
Widowed	3	0		80	15		254	47	
Divorced	38	3		323	32		108	15	
Separated	24	1		114	21		17	5	
Never married	648	18		314	28		44	9	
Living with partner	184	10		156	21		18	4	
**BMI (kg/m**^**2**^**)**			0.884			0.062			0.488
< 18.5	66	2		20	4		16	6	
18.5–24.9	612	27		562	88		254	48	
25.0–29.9	400	14		754	108		316	54	
≥ 30.0	451	18		906	97		305	52	
**Positive TST**			< 0.001			< 0.001			< 0.001
Yes	1493	42		2148	163		890	105	
No	54	21		124	135		25	57	
**Lived in household TB sick person**			0.223			0.014			0.012
Yes	28	3		85	20		30	12	
No	1507	59		2178	275		880	148	
**History of TB exposure**			0.477			< 0.001			0.009
Yes	4	1		9	13		3	4	
No	1536	61		2256	282		902	156	
**TB medicine**			0.039			0.015			0.126
Yes	21	8		43	43		15	15	
No	26	1		72	35		28	18	
Arthritis classification			0.804			0.092			0.011
No any types of arthritis	1230	55		1722	237		417	84	
Rheumatoid arthritis	5	0		123	10		70	17	
Other types of arthritis	22	1		223	21		253	27	
**Smoking status**			0.464			0.412			0.006
Current	298	17		501	56		90	29	
Former	141	5		488	69		372	67	
Never	838	34		1279	173		453	66	
**Alcohol use**			0.225			0.001			0.736
Yes	1066	35		1502	162		558	100	
No	336	15		503	86		303	51	
**Diabetes**			0.507			0.153			0.266
Yes	21	2		297	48		221	46	
No	1518	60		1916	243		660	111	

*BMI* (Body Mass Index): defined according to CDC guidelines, *TST* (Tuberculin Skin Test): induration size as positive (≥ 10 mm) and negative (< 10 mm).

Lived in household TB sick person: participant’s response to “Have you ever lived in the same household with someone while that person was sick with tuberculosis or TB?”.

History of TB exposure: participant’s response to “Were you ever told that you had active tuberculosis or TB?”.

TB medicine: participant’s response to “After getting a positive TB test, were you prescribed any medicine to keep you from getting sick with TB?”.

Arthritis classification: participant’s response to “Has a doctor or other health professional ever told you had arthritis?” and “Which type of arthritis was it?”.

Smoking status: current, having smoked 100 cigarettes and currently smoke every day or some day; former, having smoking 100 cigarettes but did not currently smoke; never, had not smoked 100 cigarettes.

Alcohol use: participant’s response to “Have you had at least 12 drinks of any type of alcoholic beverage?”.

Diabetes: participant’s response to “Other than during pregnancy, have you ever been told by a doctor or health professional that you have diabetes or sugar diabetes”.

In 18–34 age group, the QFT-GIT results in different education level (*P* = 0.018) and TB medicine groups (*P* = 0.039) showed significant difference. Both in 35–64 and 65–85 age groups, the significant difference of QFT-GIT results can be found in different gender, race, education level, lived in household TB sick person status, and history of TB exposure (all *P* < 0.05). In addition, TB medicine (*P* = 0.015) and alcohol use (*P* = 0.001) were related to QFT-GIT results in 35–64 age group. Arthritis types (*P* = 0.011) and smoking status (*P* = 0.006) were related to QFT-GIT results in 65–85 age group. Among 3 age groups, marital status, BMI, and diabetes were not associated with QFT-GIT results (all *P* > 0.05).

The comparison analysis on 23 laboratory results (Table 2) showed no significant difference between positive and negative QFT-GIT groups regarding monocyte percent, basophils percent, lymphocyte number, eosinophils number, basophils number, red blood cell count, hematocrit, PMR, PNR, and PNI (all *P* > 0.05). The remaining 11 laboratory test results presented a significant difference between the 2 groups (all *P* < 0.05).

**Table 2:** The baseline characteristics of quantitative variables stratified by QFT-GIT results (means ± SE).

	*N*	Negative	Positive	*P*
White blood cell count^#^	5250	6.98 ± 0.03	6.68 ± 0.07	0.001
Albumin (g/L)	5121	42.86 ± 0.05	42.53 ± 0.12	0.020
Sodium (mmol/L)	5119	138.93 ± 0.03	139.19 ± 0.09	0.006
Lymphocyte percent (%)	5245	30.55 ± 0.13	31.74 ± 0.31	0.001
Monocyte percent (%)	5245	7.58 ± 0.03	7.50 ± 0.08	0.436
Neutrophils percent (%)	5245	58.37 ± 0.14	57.06 ± 0.35	0.001
Eosinophils percent (%)	5245	2.82 ± 0.03	3.01 ± 0.07	0.020
Basophils percent (%)	5245	0.71 ± 0.01	0.72 ± 0.02	0.729
Lymphocyte number^#^	5245	2.07 ± 0.01	2.05 ± 0.02	0.657
Monocyte number^#^	5245	0.51 ± 0.01	0.48 ± 0.01	0.006
Neutrophils number^#^	5245	4.15 ± 0.02	3.89 ± 0.05	< 0.001
Eosinophils number^#^	5245	0.19 ± 0.01	0.20 ± 0.001	0.242
Basophils number^#^	5245	0.04 ± 0.00	0.03 ± 0.00	0.224
Red blood cell count*	5245	4.56 ± 0.01	4.57 ± 0.01	0.766
Hemoglobin (g/dL)	5245	13.90 ± 0.02	13.78 ± 0.05	0.058
Hematocrit (%)	5245	40.75 ± 0.06	40.65 ± 0.15	0.547
Platelet count^#^	5245	237.39 ± 0.904	232.36 ± 0.838	0.040
MLR	5245	0.27 ± 0.00	0.25 ± 0.00	< 0.001
PMR	5245	526.35 ± 3.84	534.58 ± 9.06	0.428
PLR	5245	126.35 ± 0.76	121.37 ± 1.61	0.015
NLR	5245	2.21 ± 0.01	2.02 ± 0.03	< 0.001
PNR	5245	66.13 ± 0.67	67.08 ± 1.01	0.589
PNI	5245	53.26 ± 0.09	52.81 ± 0.17	0.088

*BMI* body mass index; *MLR* monocyte-to-lymphocyte ratio; *PMR* platelets-to-monocyte ratio; *PMR* platelets-to-monocyte ratio; *PLR* platelets-to-lymphocyte ratio; *NLR* neutrophil-to-lymphocyte ratio; *PNR* platelets-to-neutrophil ratio; *PNI* prognostic nutritional index (*PNI* = Albumin + 5 × Lymphocyte number).

The unit for variables with a pound sign (#) was 1000 cells/uL, and for variables with an asterisk (*) was million cells/uL.

### Association of laboratory variables with QFT-GIT results

**Figure 1:**
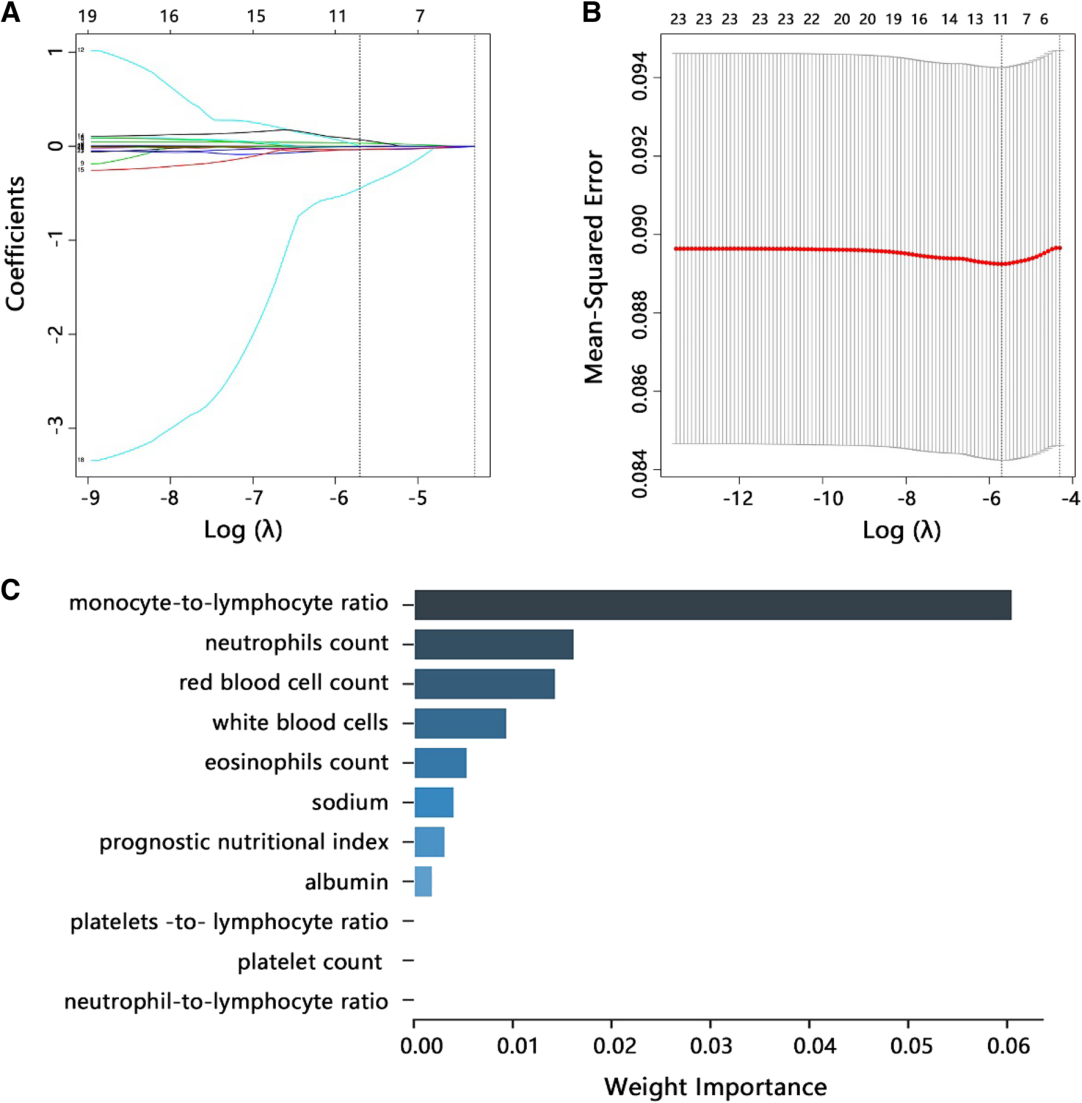
Variables selection associated with positive QFT-GIT using LASSO regression analysis. (**A**) LASSO coefficient of 23 laboratory variables. (**B**) Ten-fold cross-validation for tuning parameter selection in the LASSO model. (**C**) Weight importance evaluation of nonzero variables.

We then performed the LASSO analysis to filter the important laboratory variables associated with QFT-GIT results. Figure 1A showed the coefficients of 23 laboratory results in LASSO regression. The optimal λ value was determined by using ten-fold cross-validation (Fig. 1B). Finally, 11 variables with nonzero coefficients were identified with the optimal λ of 0.003, which included white blood cell count, albumin, sodium, eosinophils count, neutrophils number, red blood cell count, platelet count, monocyte-to-lymphocyte ratio (MLR), platelets-to-lymphocyte ratio (PLR), neutrophil-to-lymphocyte ratio (NLR), and prognostic nutritional index (PNI).

Further, we used the machine learning model based on LASSO analysis to determine the weight importance of nonzero variables (Fig. 1C). According to each variable's contribution by the machine learning model, monocyte-to-lymphocyte ratio, neutrophils number, white blood cell count, red blood cell count, and eosinophils count were the top five most important variables. Especially, the monocyte-to-lymphocyte ratio (MLR) showed the most significant association with QFT-GIT results.

We then explored the correlation between 11 variables with nonzero coefficients and QFT-GIT results by logistic regression analyses (Table. 3). The univariable logistic regression analysis showed that 9 variables were significantly related to the QFT-GIT results (all *P* < 0.05). We then enrolled the 9 variables into the multivariable analysis, indicating that sodium (OR = 1.050, *P* = 0.024) and MLR (OR = 0.188, *P* = 0.025) were independent factors of QFT-GIT results. With per 1 mmol/L sodium increase, the risk of positive QFT-GIT increased 1.050 times. With per MLR increase, the risk of positive QFT-GIT increased 0.188 times.

**Table 3:** Correlation between 11 laboratory variables and QFT-GIT by logistic regression analyses.

Covariates	Univariable		Multivariable	
	β	OR (95%CI)	β	OR (95%CI)
White blood cell count^a^	− 0.060	0.942 (0.900, 0.986)*	0.467	1.596 (0.947, 2.689)
Albumin (g/L)	− 0.038	0.963 (0.938, 0.989)**	0.106	1.112 (0.966, 1.279)
Sodium (mmol/L)	0.055	1.057 (1.013, 1.102)*	0.049	1.050 (1.006, 1.096)*
Eosinophils count^a^	0.260	1.296 (0.722, 2.328)		
Neutrophils number^a^	− 0.090	0.914 (0.862, 0.968)**	− 0.512	0.599 (0.336, 1.068)
Red blood cell count^b^	0.071	1.073 (0.896, 1.285)		
Platelet count^a^	− 0.002	0.998 (0.996, 0.999)**	− 0.001	0.999 (0.336, 1.068)
MLR	− 1.031	0.357 (0.154, 0.823)*	− 1.671	0.188 (0.043, 0.815)*
PLR	− 0.003	0.997 (0.995, 0.999)**	− 0.003	0.997 (0.990, 1.004)
NLR	− 0.151	0.860 (0.785, 0.942)**	− 0.061	0.941 (0.683, 1.297)
PNI	− 0.020	0.981 (0.962, 0.999)*	− 0.156	0.856 (0.746, 0.982)
Constant	/		− 5.259	0.005

*OR* odds ratio; *CI* confidence interval; *MLR* monocyte-to-lymphocyte ratio; *PLR* platelets-to-lymphocyte ratio; *NLR* neutrophil-to-lymphocyte ratio; *PNI* prognostic nutritional index.

The unit for variables with superscript a was 1000 cells/uL and for variables with superscript b was million cells/ uL.

*P < 0.05 and **P < 0.01.

This study further used the multivariable logistic regression analyses to estimate OR of 2 independent factors for QFT-GIT results after adjusting for potential confounders (Table. 4). The results indicated that MLR rather than sodium showed significant correlation with positive QFT-GIT (OR = 0.112, *P* < 0.001) in model 1. When this model was further adjusted for other confounders, the correlation of higher sodium level with positive QFT-GIT was significantly observed in model 2 (OR = 1.151, *P* < 0.05), model 3 (OR = 1.066, *P* < 0.05), and model 4 (OR = 1.223, *P* < 0.05). The correlation of MLR with QFT-GIT results was observed in model 3. These results indicated the significant relationship of MLR and sodium with QFT-GIT.

**Table 4:** Association of independent factors with QFT-GIT after adjusting variables.

Covariates	Sodium (mmol/L)		MLR	
	*β*	OR (95%CI)	*β*	OR (95%CI)
Crude model	0.055	1.057 (1.013, 1.102)*	− 1.031	0.357 (0.154, 0.823)*
Model 1	0.037	1.037 (0.995, 1.081)	− 2.187	0.112 (0.045, 0.281)***
Model 2	0.141	1.151 (1.016, 1.304)*	0.681	1.976 (0.160, 24.370)
Model 3	0.064	1.066 (1.014, 1.120)*	− 2.237	0.107 (0.036, 0.316)***
Model 4	0.201	1.223 (1.044, 1.433)*	0.747	2.111 (0.113, 5.403)

*OR* odds ratio; *CI* confidence interval; *MLR* monocyte-to-lymphocyte ratio.

Model 1: adjusted for age, gender, race, marital status, and education level. Model 2: adjusted for age, gender, race, marital status, education level, BMI, positive TST, lived in household TB sick person, history of TB exposure, and TB medicine. Model 3: adjusted for age, gender, race, marital status, education level, arthritis classification, smoking status, alcohol use, and diabetes. Model 4: adjusted for all variables.

### Comprehensive model construction

Due to the importance of MLR and sodium on QFT-GIT results, we further established an integrated model incorporating MLR, sodium, and clinical characteristics by nomogram analysis.

Before nomogram analysis, we firstly explored the correlation of QFT-GIT results and clinical characteristics through multivariable logistic regression analysis (Table. 5), finding that gender, age, marital status, TST, and lived in household TB sick person were independently related to the QFT-GIT results (all *P* < 0.05).

**Table 5:** Correlation of clinical variables with QFT-GIT results.

	*β*	*P*	OR (95%CI)
Gender	− 1.285	0.001	0.277 (0.131, 0.586)
Race	− 0.007	0.957	0.993 (0.770, 1.280)
Education level	− 1.171	0.435	0.842 (0.548, 1.295)
Age groups (years)	0.581	0.046	1.788 (1.011, 3.160)
Marital status	− 0.221	0.034	0.802 (0.654, 0.983)
BMI (kg/m^2^)	− 0.099	0.617	0.906 (0.614, 1.335)
Positive TST	1.473	< 0.001	4.361 (2.176, 8.739)
Lived in household TB sick person	− 1.489	0.007	0.226 (0.076, 0.672)
History of TB exposure	0.060	0.947	1.062 (0.183, 6.143)
TB medicine	− 0.332	0.325	0.717 (0.370, 1.390)
Rheumatoid arthritis	0.311	0.159	1.365 (0.886, 2.104)
Diabetes	− 0.770	0.076	0.463 (0.198, 1.084)
Alcohol use	− 0.163	0.656	0.849 (0.413, 1.744)
Smoking status	0.172	0.456	1.188 (0.756, 1.867)
Constant	5.226	0.048	6.763

Related details about these variables can be found in the Table 1.

**Figure 2:**
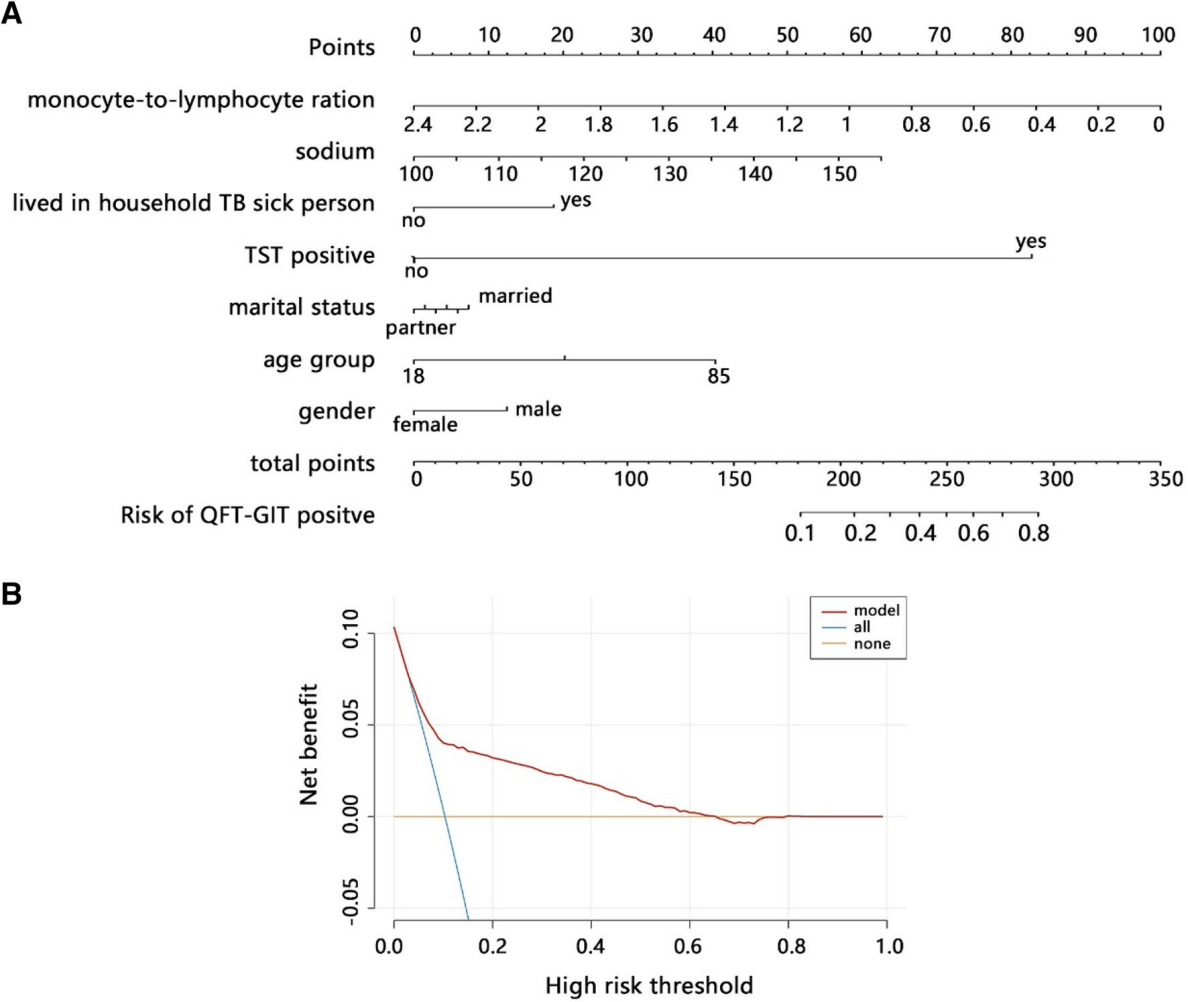
Development of the comprehensive nomogram model and its performance on QFT-GIT results. (**A**) Nomogram was constructed with the laboratory signatures and clinical variables. (**B**) Decision curve analysis (DCA) was used to assess the clinical usefulness of the comprehensive model. The x-axis indicates the threshold probability. The y-axis indicates the net benefit.

**Figure 3:**
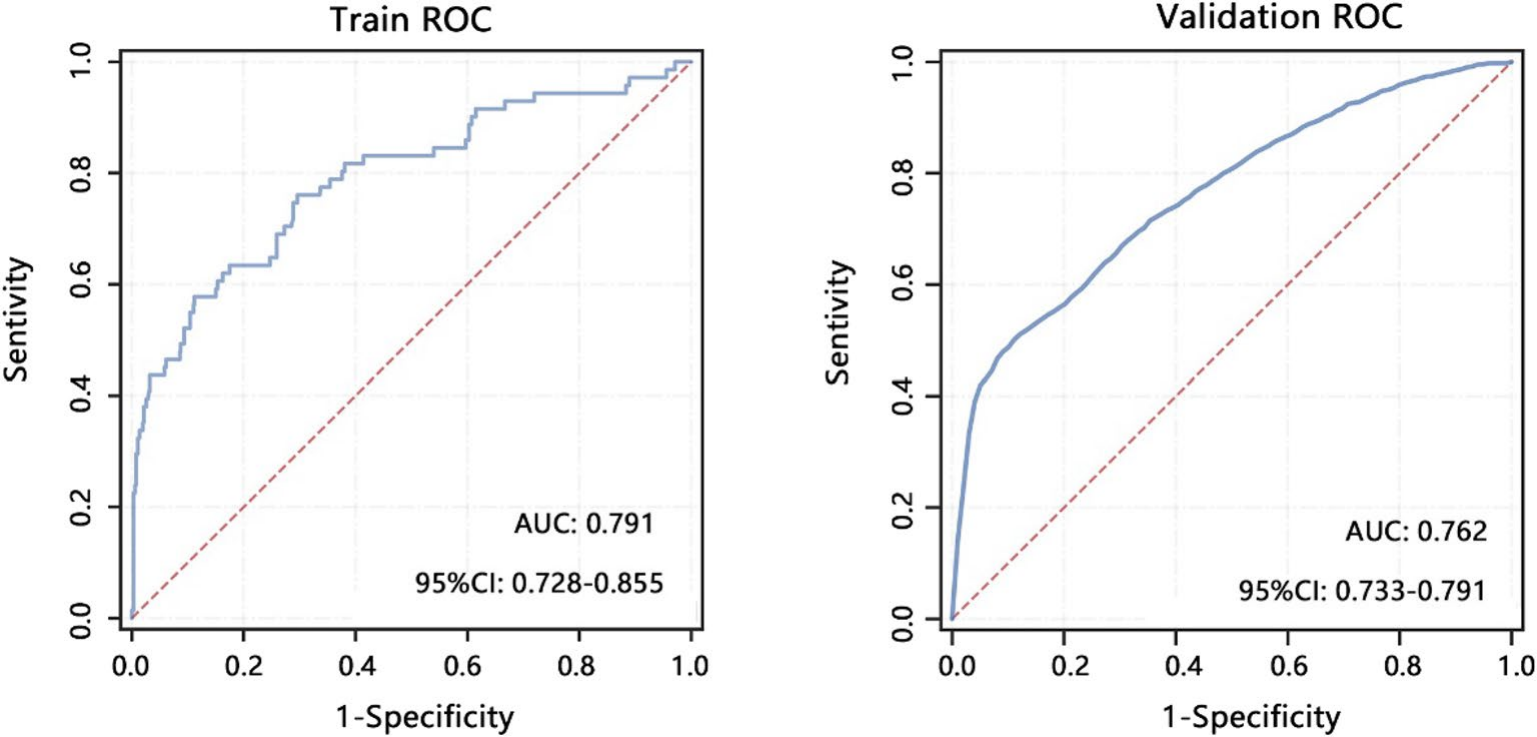
ROC analysis was used to assess the performance of the comprehensive model in training and validation sets.

Further, we integrated gender, age, marital status, TST, lived in household TB sick person, MLR, and sodium to construct a comprehensive nomogram model. The nomogram analysis (Fig. 2A) indicated that the comprehensive model can predict 0.8 probability for positive QFT-GIT, and MLR presented the largest contribution to the GFT-GIT in this model. The DCA analysis indicated that comprehensive model achieved more net benefit than either treat-all-positive or treat-none-positive strategies across all ranges of threshold probability (Fig. 2B). In addition, the ROC analyses (Fig. 3) both in training set (AUC = 0.791) and validation set (AUC = 0.762) showed that comprehensive model had favorable performance to predict positive QFT-GIT.

### Correlation between variables and mortality risk in individuals with QFT-GIT positive

**Figure 4:**
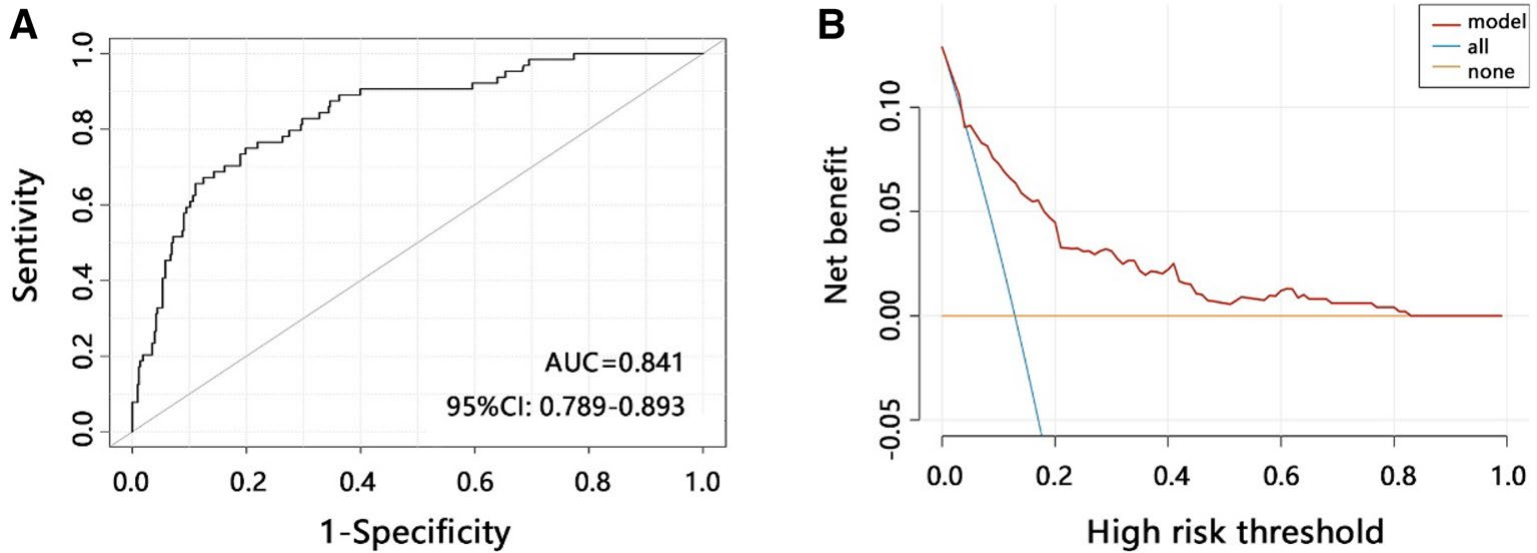
Development of the comprehensive model and its performance on death risk in individuals with positive QFT-GIT. (**A**) ROC curve analysis was used to predict death. (**B**) Decision curve analysis (DCA) was used to assess the clinical usefulness. The x-axis indicates the threshold probability. The y-axis indicates the net benefit.

We then explored the potential of comprehensive model to predict the disease development of individuals with positive QFT-GIT. Through ROC analysis, we also found a favorable performance of comprehensive model to predict death (Fig. 4A, AUC = 0.841). The DCA analysis indicated that comprehensive model achieved more net benefit across all ranges of threshold probability (Fig. 4B).

In addition, we further assessed the significant correlation of MLR and sodium themselves with death risk in individuals with positive QFT-GIT (Table. 6). The correlation of sodium with death was not observed in both crude model and adjusted models. However, the MLR showed significant correlation with death risk in crude model and other adjusted models (all *P* < 0.05). These results further confirmed the importance of MLR on the disease development among individuals with positive QFT-GIT.

**Table 6:** Association of independent factors with death using multivariate logistic regression.

Covariates	Sodium (mmol/L)		MLR	
	*β*	OR (95%CI)	*β*	OR (95%CI)
Crude model	0.029	1.029 (0.917, 1.155)	5.545	9.743 (3.690, 19.289)***
Model 1	0.002	1.002 (0.888, 1.130)	4.716	7.655 (1.523, 13.743)**
Model 2	0.208	1.231 (0.871, 1.740)	9.173	7.873 (2.562, 13.238)**
Model 3	0.049	1.050 (0.883, 1.250)	6.600	5.275 (2.489, 12.788)***
Model 4	0.192	1.211 (0.796, 1.844)	2.872	4.253 (1.985, 9.783)*

*OR* odds ratio; *CI* confidence interval; *MLR* monocyte-to-lymphocyte ratio.

Model 1: adjusted for age, gender, race, marital status, and education level. Model 2: adjusted for age, gender, race, marital status, and education level, BMI, positive TST, lived in household TB sick person, history of TB exposure, and TB medicine. Model 3: adjusted for age, gender, race, marital status, education level, arthritis classification, smoking status, alcohol use, and diabetes. Model 4: adjusted for all variables.

## Discussion

This study evaluated the correlation of 23 laboratory variables with positive QFT-GIT, finally finding that MLR (monocyte-to-lymphocyte ratio) and sodium were independent factors associated with QFT-GIT result. As the level of MLR increased, the risk of positive QFT-GIT decreased. Our study disclosed the importance of MLR in TB infection. Presently, more and more researchers have paid more attention to the MLR as a biomarker. For example, Cheng et al. found that MLR was significantly associated with an increased risk of depression^19^. Huang et al. found that MLR was associated with a 2-year relapse in patients with multiple sclerosis^20^. Kamiya et al. found that MLR can be a helpful diagnostic marker for lymphoma in adults with peripheral lymphadenopathy when the etiology is unclear^21^. The potential clinical value of MLR have been disclosed in various disease.

At present, previous studies have reported the significant role of MLR in TB-related disease. Chen et al. study showed that MLR was an independent factor in the diagnosis of spinal TB and was associated with the severity of spinal TB^22^. Choudhary et al. study found that MLR was related to TB and declined with anti-TB treatment in HIV-infected children^23^. Gatechompol et al. study indicated that increased MLR can predict the incident TB among people living with HIV on or after antiretroviral therapy, and at cut-point 0.23, the MLR provided a diagnostic AUC of 0.849 and a sensitivity of 85%, and specificity of 71%^24^. Sukson et al. study showed that MLR > 0.45 was the best cut-off point for diagnosing TB pleuritis where the sensitivity and specificity were 82.5% and 86.3%, respectively^25^. These researches have disclosed the significant clinical value of MLR in TB-related diseases, and its significance in TB needs more investigation.

We continuously explored the relationship between MLR and mortality risk in individuals with positive QFT-GIT, indicating that MLR was also an important prognosis predictor after adjusting potential confounders. As the level of MLR increased, the mortality risk of individuals increased. Circulating monocytes are first recruited to the infection sites and are induced to differentiate into M2 macrophages, acquiring the suppression function on adaptive immune response^26^. While lymphocytes are crucial for the adaptive immune response. The decrease of lymphocytes' absolute count may reflect an insufficient response of the host immune system to the disease, consequently enhancing the disease progression^27^. MLR has been referred to as an inflammatory and immune-suppressive index. Olivia et al. study showed that active TB individuals had a significantly higher level of MLR compared to both latent TB and no latent TB individuals^28^. It followed that MLR level was related to the severity of TB. In this study, a higher MLR value predicted the higher mortality risk of positive QFT-GIT samples. We speculated that positive QFT-GIT individuals might be constantly exposed to Mtb infection, which caused a constant inflammatory status in infection sites, thus deteriorating the TB progression. Hence, regulating the dysregulation of host immune response might contribute to the control of tuberculosis progression.

Finally, our study was subject to limitations. This study defined the tuberculosis infection as positive QFT-GIT, which may cause the problems of false positives even if the specificity of an IGRA were 95%. Dorman et al. found that most conversions among healthcare workers in low TB incidence settings appeared to be false positives, and these occurred 6 to 9 times more frequently with IGRAs than TST^29^. Mancuso et al. found that in low-prevalence populations, most discordance between different tests for latent tuberculosis infection, can be interpreted by false-positives^30^. In addition, our study was a cross-sectional design, and as such we were unable to determine the temporal relationship between factors and TB.

## Conclusions

This study enrolled 5256 individuals into analysis, of which 521 had the positive QFT-GIT. Through LASSO and logistic regression analyses, sodium and MLR were identified to be independently associated with QFT-GIT result among 23 laboratory variables. After adjusting for potential confounders, the correlation between them was still observed. Based on MLR, sodium, and significant clinical characteristics, we constructed a comprehensive nomogram model, finding that comprehensive model had favorable performance for predicting QFT-GIT result and death risk of individuals with positive QFT-GIT. Further analysis showed that MLR rather than sodium was independently related to the death risk. Our study suggested that MLR might be an important factor in the initiation and progression of TB.

## Data Availability

Related data were publicly available from the NHANES at https://www.cdc.gov/nchs/nhanes/. The survival condition of participants in the cases of participants above 18 years old was available from NDI at https://www.cdc.gov/nchs/ndi/index.htm.
